# Results of the first interim analysis of the *RAPPER* II trial in patients with spinal cord injury: ambulation and functional exercise programs in the REX powered walking aid

**DOI:** 10.1186/s12984-017-0274-6

**Published:** 2017-06-19

**Authors:** Nick Birch, Jon Graham, Tom Priestley, Chris Heywood, Mohamed Sakel, Angela Gall, Andrew Nunn, Nada Signal

**Affiliations:** 1 Consultant Spinal Specialist, The Chris Moody Rehabilitation Centre, Moulton College, Northampton, NN3 7QL UK; 2 Consultant Physiotherapist, PhysioFunction Ltd, The Chris Moody Rehabilitation Centre, Moulton College, Northampton, NN3 7QL UK; 3 Research Manager, Rex Bionics PLC, 4th Floor, 1-3 Pemberton Row, London, EC4A 3BG UK; 4 Clinical Research Fellow, The Chris Moody Rehabilitation Centre, Moulton College, Moulton, Northampton, NN3 7QL UK; 5 Consultant Physician and Director of NeuroRehabilitation, East Kent Hospitals University NHS Foundation Trust, Ethelbert Road, Canterbury, CT1 3NG UK; 6 Consultant, London Spinal Cord Injury Centre, Royal National Orthopaedic Hospital, Brockley Hill, Stanmore, Middlesex, HA7 4LP UK; 7 Medical Director, Victorian Spinal Cord Service, Austin Hospital, 145 Studley Road, PO Box 5555, Heidelberg, VIC 3084 Australia; 8 Senior Research Fellow, AUT University, Private Bag 92006, Auckland, New Zealand

**Keywords:** Spinal cord injury, Tetraplegia, Paraplegia, Powered walking aid, Assistive technology, Physiotherapy, Rehabilitation, Robotics

## Abstract

**Background:**

The RAPPER II study investigates the feasibility, safety and acceptability of using the REX self-stabilising robotic exoskeleton in people with spinal cord injury (SCI) who are obligatory wheelchair users. Feasibility is assessed by the completion of transfer into the REX device, competency in achieving autonomous control and completion of upper body exercise in an upright position in the REX device. Safety is measured by the occurrence of serious adverse events. Device acceptability is assessed with a user questionnaire.

**Methods:**

RAPPER II is a prospective, multi-centre, open label, non-randomised, non-comparative cohort study in people with SCI recruited from neurological rehabilitation centres in the United Kingdom, Australia and New Zealand. This is the planned interim report of the first 20 participants. Each completed a transfer into the REX, were trained to achieve machine control and completed Timed Up and Go (TUG) tests as well as upper body exercises in standing in a single first time session. The time to achieve each task as well as the amount of assistance required was recorded. After finishing the trial tasks a User Experience questionnaire, exploring device acceptability, was completed.

**Results:**

All participants could transfer into the REX. The mean transfer time was 439 s. Nineteen completed the exercise regime. Eighteen could achieve autonomous control of the REX, 17 of whom needed either no assistance or the help of just one therapist. Eighteen participants completed at least one TUG test in a mean time of 313 s, 15 with the assistance of just one therapist. The questionnaire demonstrated high levels of acceptability amongst users. There were no Serious Adverse Events.

**Conclusions:**

This first interim analysis of RAPPER II shows that it is feasible and safe for people with SCI to use the REX powered assisted walking device to ambulate and exercise in. Participants with tetraplegia and paraplegia could walk and perform a functional exercise program when standing needing only modest levels of assistance in most cases. User acceptability was high.

**Trial registration:**

ClinicalTrials.gov, NCT02417532. Registered 11 April 2015.

## Background

The World Health Organisation estimates that the annual global incidence of Spinal Cord Injury (SCI) is between 40 and 80 cases per million population [1]. Of all the functional impairments following SCI, loss of ambulatory capacity ranks as one of the highest concerns in affected people [2]. Devices that can restore ambulation are therefore of great interest to this patient population and may bestow potential health benefits.

The first designs of an assisted walking device or “exoskeleton” for people with paralysis were patented in the late nineteenth century [3], but it has taken more than a century of effort to produce workable robotic exoskeletons that may have clinical utility. In that time, two divergent technological paradigms have emerged to enable people with complete or near complete paralysis to ambulate. One is characterised by robotic exoskeletons that require the user to supplement their balance with crutches or a walking frame (the *Four Point Walking Devices*). Examples of this include ReWalk (ReWalk Robotics, Yokneam, Israel), Ekso (Ekso Bionics, Richmond CA, USA) and Indego (Parker Hannifin, OH, USA), The other is REX (Rex Bionics PLC, London, UK); a self-stabilising robotic exoskeleton which requires no supplemental upper body support to balance.

The advantage of the Four Point Walking Devices is speed [4], but the need to use supplementary walking aids, such as crutches, to balance and change direction means that they interfere with normal upper body function. In addition, they are less suitable for people with tetraplegia and higher thoracic lesions and they are generally recommended for people with paraplegia and incomplete lesions [5]. The REX, in comparison, ambulates slowly but has powered manoeuvrability in multiple directions including forwards, backwards and side-stepping and is self-stabilising so users need no further external support, leaving the upper body relatively free for other functions. The device does not currently have the facility to ascend and descend stairs. It is suitable for those with cord / conus lesions from C4 to L5, meaning that people with tetraplegia and paraplegia can use it with almost equal facility, irrespective of lesion completeness [6]. Common to both types of device, users can function to varying degrees in un-adapted environments, which can have a positive psychosocial impact.

**Fig. 1:**
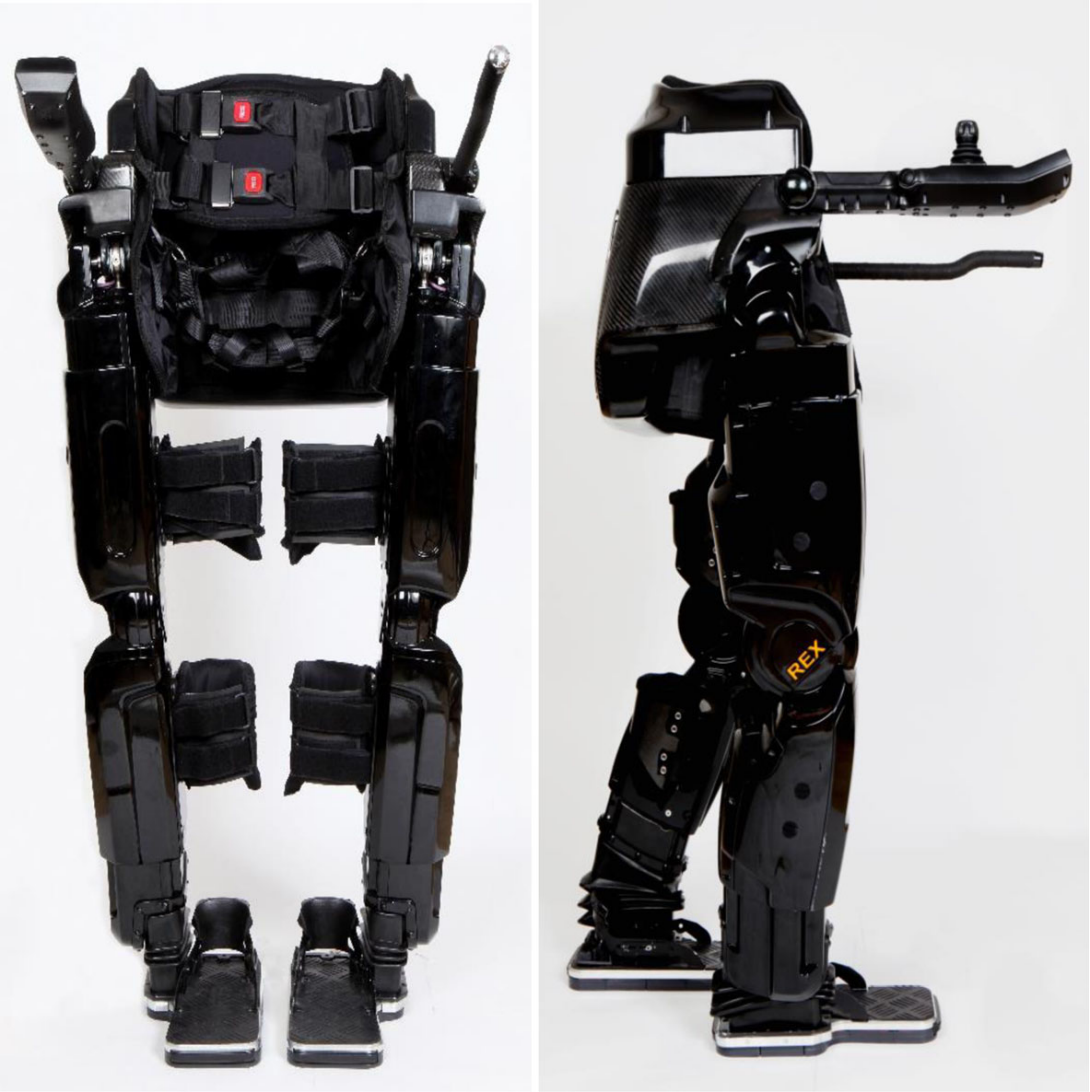
REX robotic exoskeleton seen from the front and side

In the REX device, a trained user can move from sitting to standing, step forwards, backwards and sideways, lean (weight-shift) left and right, walk forward and backwards, and turn in both directions on flat, smooth, dry surfaces. The REX, with an adjustment mechanism for lower limb length, is designed to fit a range of body sizes and be used under the supervision of a qualified Health Care Professional [7]. The REX-P product, with a fixed limb length setting for a single user, is designed for use “At Home” with a trained “Buddy” in attendance [8]. The thigh and shin cuffs maintain the alignment of the legs in relation to the device’s articulations and a harness attached rigidly to the frame of the machine supports the trunk and pelvis. (Fig. 1). Robotics include sensors with high frequency sampling to continuously detect the location of the moving parts, governed by microprocessors running proprietary computer code controlling 10 linear actuators (two at each articulation of the device corresponding to the user’s hip and ankle, and one corresponding to the knee). Human control is through a joystick and a three-button keypad, similar in design to many gaming devices and an LCD screen showing the simple hierarchical menus. Power is provided by a rechargeable, interchangeable lithium-polymer battery which when fully charged allows 120 min of continuous use [7].

The impairment of motor and sensory function below the level of the lesion, and consequent loss of ambulatory function following SCI results in an extreme form of deconditioning [9]. Consequent disruption to the cardiovascular, musculoskeletal, nervous, digestive, integumentary, renal and respiratory systems can cause significant long term health problems [10]. Ambulatory physical activity and exercise may represent one of the most potent methods of counteracting the negative effects of deconditioning in SCI [9]. In people with SCI physical activity and exercise has been shown to offer both physical and psychological benefits [9, 11]. There is good evidence to support its positive effects on cardiorespiratory fitness and muscle strength, with emerging evidence of its effect on mood, spasticity, bladder and bowel function [12]. International experts in SCI rehabilitation including the American College of Sports Medicine and the American Physical Therapy Association recommend that people with SCI undertake regular cardiovascular, endurance and strength training [9, 12–14]. However, identifying suitable methods, and overcoming barriers to engaging in exercise and physical activity represents a significant challenge in this population [13, 15]. Robotic exoskeletons offer the possibility for people with SCI to engage in physical activity and exercise and they may enable a range of exercise activities to be undertaken, dependent on the functionality and stability of the exoskeleton, and the level and extent of an individual’s SCI [13]. Self-stabilising powered exoskeletons which require no supplemental upper body support to balance, such as REX, may enable the user to undertake not only ambulatory exercise, but also upper body exercise in an upright position [6, 7].

Four key priorities have previously been identified as being important in the future development of exoskeletons: robust control, safety and dependability, ease of wearability (or portability) and usability/acceptance [16]. Although REX fulfils these criteria, such priorities do not necessarily capture the full range of concerns of the stakeholders in this area of rapidly advancing technology, who include the users, their primary/domestic caregivers and healthcare professionals involved in their care. When directly surveyed, some of the features of robotic assisted ambulation that were identified as being most important included: specific and general health benefits, comfort and safety of the devices and functionality that allowed tasks to be carried out in a standing position [17]. Studies involving Four Point Walking devices have largely concentrated on their role as an assistive device, emphasising speed and the physiological efficiency of walking [4, 18, 19]. The RAPPER II trial directly addresses a number of the key priorities identified by stakeholders, by investigating feasibility, safety and acceptability of the REX device when used to undertake ambulatory physical activity and upper body exercise in people with spinal cord injury.

## Methods

RAPPER II is a prospective international, multi-site, open label, non-randomised, non-comparative, observational registry study of Robot-Assisted Physiotherapy Exercises with the REX robotic exoskeleton in people with SCI which precludes unsupported ambulation. The objective of the study is to evaluate feasibility, safety and acceptability of the REX device when used for the first time by people with paralysis in SCI Hospitals or Rehabilitation Centres under the supervision of a physician and/or qualified rehabilitation specialist.

It is registered on the ClinicalTrials.gov website (ClinicalTrials.gov identifier: NCT02417532) and has ethics committee approval from the UK NHS Health Research Authority (NRES Committee East Midlands – Derby: REC Number – 15/EM/0196) in accordance with the Declaration of Helsinki (59th WMA General Assembly, Seoul, October 2008) and the principles of Good Clinical Practice (GCP). Oversight of the trial is provided by the independent Clinical Research Organisation: Generic Devices Consulting, Inc. Florida, US.

The primary outcomes of the trial are: completion of transfer into the REX device, completion of upper body exercises in an upright position in the REX device, and adverse events.

The secondary outcomes are: time to complete the transfer, competency in achieving autonomous control of the REX device, the Timed Up and Go (TUG) test, the level of assistance required to complete each task or group of tasks and device acceptability as assessed by a user questionnaire.

A record of any unexpected Serious Adverse Events (SAE) within trial episodes is kept. These are defined as death, a life threatening adverse event or an event occurring as a result of the use of the device that requires medical intervention. Any death as a result of participation in the trial results in cessation as do any three other serious adverse events.

**Fig. 2:**
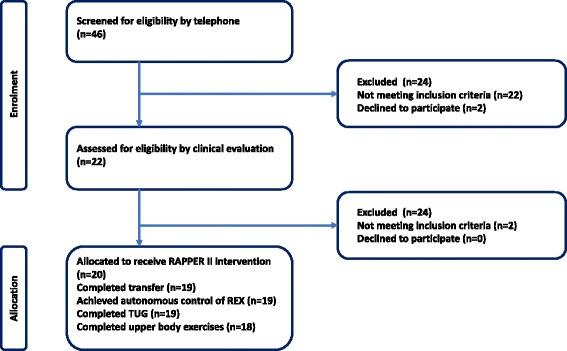
Study Flowchart

The study procedures and participant recruitment are outlined in Fig. 2. Following ethics committee approval there was wide dissemination of information about the trial through local clinical practices, national SCI organizations, television and social media. Screening is performed telephonically through a single point of contact in each participating country. Once a person becomes a candidate for enrolment they have an appointment with a research investigator at their nearest participating trial centre. Potential participants are provided with a two-part patient information document detailing the purpose of the study and what to expect if they take part, including potential risks. Potential participants are then provided with written informed consent and are subsequently clinically assessed against the inclusion and exclusion criteria. In brief, participants are included in the study if they have a SCI (C4 to L5), are obligatory wheelchair users, have no contraindications to standing and walking in the REX device and meet the anthropometric requirements of the device (between 1.42m and 1.93m in height, 40 kg to 100 kg in weight, sufficient lower limb passive range of motion and manual dexterity to operate a T-bar joystick controller (7). Refer to the trial protocol for a detailed description of the inclusion and exclusion criteria (https://clinicaltrials.gov/ct2/show/NCT02417532). Potential participants who meet the inclusion criteria are then enrolled in the trial.

Within a single session, that lasts between three and four hours, eligible participants are trained in device use and then control the device to undertake ambulatory tasks and upper body exercise. Participants first transfer into the REX. This process is timed and the amount of assistance required is recorded. If a hoist is required to transfer this is also recorded. Following transfer the participant is appropriately positioned and secured in the device; trunk, pelvis and limb positions are checked for correct alignment before any movement is allowed. Participants are then trained to use the T-bar joystick and menus, and once comfortable they can independently control the device. Autonomous control of the device is deemed to be achieved if the participant can stand up, lean (weight shift) to the left and right, step forward and backwards and sit down. The time taken for instruction and the time taken to perform these activities is recorded.

Participants then perform a practice TUG test [20]. The TUG test involves rising from sitting, walking forward three metres, turning, walking back and returning to a seated position. Two further TUG tests are conducted and the times are recorded.

Lastly, two upper body exercises are undertaken in an upright position; bilateral shoulder abduction (Exercise 1) followed by lateral trunk extension to the left and right (Exercise 2). Each exercise is repeated three times and the degree of assistance required is recorded. The level of assistance needed for all tasks is defined as being either independent, supervised (no actual physical assistance needed) or requiring between one and three assistants. Following completion of the training session skin integrity and lower limb passive range of motion is re-assessed. In the event of any concerns or adverse events participants are followed up within 24 hours and thereafter as required.

At the end of the session the participants complete a 16-point questionnaire, which addresses aspects of device acceptability including ease of transfer, safety, stability, ease of control, comfort, size, sound, speed and general acceptability of the device. Participants rate statements on a 7 point Likert scale where; 7 = strongly agree, 4 = neutral, 1 = strongly disagree.

Descriptive statistics are used for all primary and secondary endpoints with 95% confidence intervals (95% CI) as appropriate using SPSS 13.0 software (SPSS Inc., Chicago, USA). The analysis has primarily been carried out on an intention to treat (ITT) basis. Differences in outcomes between those with tetraplegia and paraplegia and those with complete and incomplete lesions are also presented.

## Results

Demographic details of the first 20 participants are shown in Table 1. Fourteen men and six women had been recruited into the study by the first planned interim analysis point. The mean age was 40.9 years with a wide range (19–65 years; 95% CI +/− 5.8 years). The mean time since injury was 8.1 years with a range of 1–52 years (95% CI +/− 5.0 years).

**Table 1:** Demographics of Study Participants (*N* = 20)

Gender	Male	14
	Female	6
Age (yrs)	Mean +/− 95% CI	40.9 +/− 5.8
	Range	19–65
Time since SCI (years)	Mean +/− 95% CI	40.9 +/− 5.8
	Range	1–52
Injury level	Tetraplegia (C4-C8)	5
	Paraplegia (T1-L5)	15
Injury extent	Incomplete (ASIA B-D)	9
	Complete (ASIA A)	11

**Fig. 3:**
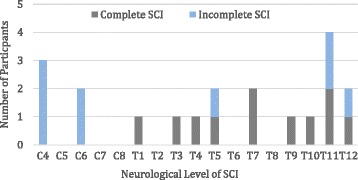
Distribution of spinal cord Injury levels and extent in the study population

Three quarters of the participants had paraplegia (cord injury levels between T1 and L5) and a quarter had tetraplegia (cord injury levels between C4 and C8). The five participants with cervical level injuries all had incomplete lesions. Eleven participants with paraplegia had complete cord injuries and four incomplete injuries. The distribution of injury level and extent is illustrated in Fig. 3.

**Fig. 4:**
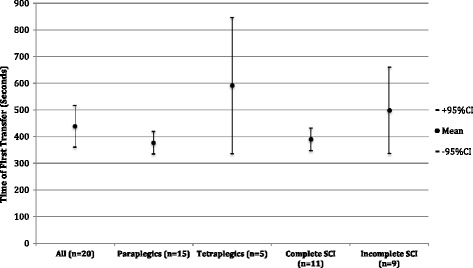
Time of Transfer in First Use - seconds (Mean +/− 95% Confidence Intervals)

All 20 participants could transfer into the device (Table 2), but there was considerable variation in the time to transfer with a mean for the whole group of 439 seconds (range 230 – 1007 seconds; 95% CI +/- 78 seconds). Subgroup analysis showed least variation in the groups with paraplegia and complete injuries (Fig. 4). The participants with cervical and incomplete injuries had longer mean transfer times, but there were wide ranges and large confidence intervals.

**Table 2:** Transfer in First Use - Time and Levels of Assistance required

		Mean Time (Range): seconds	Supervised	1 Assistant	2 Assistants	3 Assistants	Hoist
Total	(*n* = 20)	439 (230–1007)	4	6	8	0	2
Injury level	Tetraplegia (*n* = 5)	591 (350–1007)	0	1	2	0	2
	Paraplegia (*n* = 15)	388 (230–538)	4	5	6	0	0
Injury extent	Complete (*n* = 11)	390 (292–490)	2	3	6	0	0
	Incomplete (*n* = 9)	499 (230–1007)	2	3	2	0	2

Four participants could transfer into the REX with no actual assistance needing just supervision, six required one assistant and eight needed two assistants. Two participants with C4 lesions required hoist transfers.

Nineteen participants completed both sets of upper body exercises (Table 3). One participant was found to have such a degree of spinal deformity following prior spinal surgery that once in a standing position in the device, they required both hands to support the trunk and, even with assistance, an upright position could not be achieved. The extent of the deformity was not fully apparent when the participant was in a wheelchair. As a result, this participant could not undertake either set of upper body exercises nor did they achieve autonomous control of the device or complete the TUG.

**Table 3:** Upper Body Exercise - Levels of Assistance required

		Exercise 1 (Bilateral shoulder abduction)				Exercise 2 (Lateral Trunk Extension)			
		Supervised	1 Assistant	2 Assistants	Unable to Complete	Supervised	1 mAssistant	2 Assistants	Unable to Complete
Total	(*n* = 20)	13	5	1	1	12	5	2	1
Injury level	Tetraplegia (*n* = 5)	2	2	1	0	0	3	2	0
	Paraplegia (*n* = 15)	11	3	0	1	12	2	0	1
Injury extent	Complete (*n* = 11)	8	2	0	1	9	1	0	1
	Incomplete (*n* = 9)	5	3	1	0	3	4	2	0

The level of assistance required to complete upper body exercises was low, with 18 participants able to do bilateral shoulder abduction and 17 participants could do lateral trunk extension to the left and right, with either no help or just one assistant.

One participant had significant truncal shift and could do lateral trunk extension on the left with no assistance, but needed assistance on the right. For the purposes of this analysis this participant was counted as needing one assistant for the lateral trunk extension exercise.

Participants with paraplegia and complete spinal cord lesions needed less assistance than those with tetraplegia or incomplete lesions. Despite the level of their SCI, three out of the five participants with cervical lesions managed both exercises with either no help or just one assistant.

Eighteen participants achieved autonomous control of the device. The participant with a severe spinal deformity (see above) lacked adequate trunk control to be able to position the right upper limb to use the joystick. One participant with a C4 incomplete lesion could control the T-bar joystick in backwards and forwards movements, but could not rotate through the menus.

Nineteen participants could complete the TUG tests (Table 4) in a mean time of 313 seconds (95% CI +/- 27.9 seconds). There was very little variation between the subgroups. Fifteen participants needed the help of one assistant and four need two assistants to complete the tests.

**Table 4:** Timed Up and Go (TUG) test - average time and levels of assistance required

		Average Time (+/− 95% CI): seconds	1 Assistant	2 Assistants	Unable to Complete
Total	(*n* = 20)	313 (+/−27.9)	15	4	1
Injury level	Tetraplegia (*n* = 5)	302 (+/− 49.6)	3	2	0
	Paraplegia (*n* = 15)	317 (+/− 35.4)	12	2	1
Injury extent	Complete (*n* = 11)	324 (+/− 39.3)	9	2	0
	Incomplete (*n* = 9)	298 (+/− 39.2)	6	2	1

Regarding the physical safety of participants using the REX, there were no device related adverse events as defined in the trial protocol. In addition, visual inspection of the areas sensitive to pressure in each participant following the intervention, showed no signs of redness or bruising. There were no cases of late notification of any adverse events in this group of participants.

Nineteen participants completed the questionnaire about device acceptability (Table 5). The participant with the severe spinal deformity declined to because insufficient time had been spent in the robot for a valid evaluation to be made. A better than 80% positive/neutral response was recorded for 15 of the 16 statements after a single session (Table 1). Eighteen of 19 (95%) of the responses to Question 2 (*“I felt very confident in REX*”) and Question 4 *(“I felt very stable in REX”*) were positive. 17 participants (89%) felt safe (Question 3).

**Table 5:** Results of Device Acceptability Questionnaire (*n* = 19)

	Strongly disagree (1)	Somewhat disagree (2)	Disagree (3)	Neutral (4)	Agree (5)	Somewhat agree (6)	Strongly agree (7)	Median Rating
1. I found it easy to transfer into REX	0	3	6	1	2	4	3	disagree/neutral
2. I felt very confident in REX	0	0	0	1	3	2	13	somewhat agree/strongly agree
3. I felt very safe in REX	0	1	0	1	2	3	12	somewhat agree/strongly agree
4. I felt very stable in REX	0	1	0	0	5	3	10	somewhat agree/strongly agree
5. REX was easy to control	2	0	0	1	6	3	7	agree/somewhat agree
6. I found REX comfortable	0	0	0	4	3	0	12	somewhat agree/strongly agree
7. I enjoyed my experience in REX	0	0	0	1	3	0	15	somewhat agree/strongly agree
8. I would like to use REX on a weekly basis	0	0	1	0	3	0	15	somewhat agree/strongly agree
9. I would recommend REX to a friend	0	0	0	3	1	0	15	somewhat agree/strongly agree
10. I felt a sense of wellness after using REX (mentally or physically)	0	0	0	4	2	2	11	agree/somewhat agree
11. REX exceeded my expectations	0	0	1	3	3	4	8	agree/somewhat agree
12. The size of REX did not bother me	0	1	3	2	5	2	6	agree
13. The sound of REX did not bother me	0	2	2	1	8	1	5	agree
14. The speed of REX was suitable for me	0	1	1	2	8	4	3	neutral/agree
15. I can see the benefits of using REX regularly	0	0	0	0	4	4	11	somewhat agree/strongly agree
16. I would like to see REX more accessible to those who need it	0	0	0	1	1	0	17	somewhat agree/strongly agree

Across the group the answer to only one question (“*I found it easy to transfer into the REX”*) produced equivocal responses.

Sub-group analysis showed no difference in the rate of positive and negative responses between people with paraplegia and those with tetraplegia, nor was there a difference between people with complete SCI lesions and those with incomplete lesions.

## Discussion

This first planned interim analysis of the results of the RAPPER II trial reports on the largest number of people with SCI using a self-stabilising robotic exoskeleton ever systematically studied. The findings of this study indicate that the REX is feasible and safe to use for ambulatory physical activity and upper body exercise in a rehabilitation environment in people with chronic SCI. The primary and secondary outcomes demonstrate that for people with SCI, transfer into, achieving autonomous control of, and exercising within the REX, even when they have never used the device previously, is achievable within a single session.

Transfer into the REX in first use was speedy for some of the participants with complete thoracic cord lesions, but slower for others particularly those with high cervical lesions who needed a hoist. This was reflected in users’ evaluation of device acceptability. However, experience in clinics where REX is used for rehabilitation demonstrates that users frequently become adept at transfer relatively quickly and the level of acceptability of the device rises as a result.

The relative simplicity in achieving control of the REX was demonstrated by the low level of assistance required to use the device for most participants, the short time required to achieve autonomous control and positive user evaluation of confidence, sense of safety, stability and ease of control when using the REX. This likely reflects the simple controls and menus and the limited manual dexterity required to use the T-bar joystick. User acceptability of new technologies depends to a large part on the ease with which the controls can be mastered [21, 22]. This initial report from RAPPER II shows that REX fulfils this criterion.

Upper body exercises were completed by 19 of the 20 participants, including those with tetraplegia. This indicates that self-stabilising powered exoskeletons such as the REX, which require no supplemental upper body support to balance, offer a broad range of people with SCI the opportunity to exercise their upper bodies in an upright position. This potentially extends the case for use of robotic exoskeletons beyond assistive devices for ambulation. Future research should explore the types of exercise which can be feasibly undertaken in the REX device; the utility of the device to engage with exercise equipment originally designed for able bodied users and the physiological demands of exercising in this manner. Because of the small numbers in the sub-groups of this interim report the analysis is highly skewed and the specific utility of the REX for people with complete and incomplete lesions at different levels of the spinal cord cannot be stated at this juncture. A further RAPPER II trial report with larger numbers in the relevant sub-groups will be able to address this question with more certainty.

Nineteen of the 20 participants could undertake a suite of ambulatory tasks including walking, turning and sit-to-stand in the REX device. Whilst ambulation in REX is slow compared to the Four Point Walking devices, the REX extends the possibility of upright mobility to a broader range of people with SCI, including those with cervical lesions. The physiological demands associated with ambulation in self-stabilising powered exoskeletons such as the REX have yet to be established, however research suggests that a cardiovascular load is likely and may be dependent on the lesion level and extent and the speed of movement [14]. To date there has been limited research investigating the role of robotic exoskeletons in the rehabilitation of ambulatory functions, with much of the work focusing on their role as assistive devices [23, 24]. In some respects, ambulation in the REX device has similarities to robot assisted body weight supported treadmill training in which walking is possible because the trunk and pelvis are supported during gait rehabilitation [11]. Further exploration of the rehabilitative and health benefits of ambulation in robotic exoskeletons is required.

The level of assistance needed to undertake upper body exercise and ambulatory tasks was low and as a result there may be positive implications for rehabilitation resource allocation in the future. If rehabilitation clinicians know that patients' rehabilitation and exercise programs can be effectively managed without high staff requirements, they might consider adopting this type of technology. Given that this trial investigated a single session with users new to the technology, it may be suggested that supervisory and assistive demands would reduce over time as users became more confident using the REX device.

At this stage of the trial, with limited numbers, which may not reflect the whole SCI population, there is no clear difference between the results of people with cervical level spinal injuries and those with thoracic level injuries. Nor are there any obvious differences between the results of people with complete and incomplete spinal cord injuries. Consequently, in this cohort REX has been shown not only to be equally safe and feasible for both sub-groups but also unique amongst the robotic exoskeletons, since the Four Point Walking devices that aid mobility are not compatible with spinal cord lesions in the high- to mid-cervical spine [5].

Features of robotic exoskeletons that have been identified as important to users and their care givers include general health benefits, comfort, safety and functionality that allows daily tasks to be carried out in the standing position [16, 17]. This study has demonstrated that most participants felt safe, confident and comfortable in the REX when undertaking ambulatory tasks and upper body exercises in standing.

Limitations of this study include the relatively small sample size, the necessarily limited range of upper body exercises that could be undertaken by first time users in one session and the lack of post intervention follow-up. Also as an open label study there is probable selection bias since only highly motivated people volunteered for and participated in the study.

The next planned interim analysis will be of the first 50 recruits in the RAPPER II trial and it will address the sample size and follow-up issues. The larger number of participants will further enable exploration of whether there are differences between people with cervical and thoracic lesions or those with complete and incomplete spinal cord lesions. In addition, a protocol update introduced after the first 20 participants were enrolled (agreed with the Ethics Committee as a Major Protocol Amendment) has added pre- and post-trial evaluation of quality of life, spasticity, sleep and pain to the reporting schedule. The second report of the RAPPER II trial will therefore explore patient reported outcomes following REX use.

## Conclusions

REX, as a potential rehabilitation tool in people with SCI, has been shown by the results of this first planned interim report of the RAPPER II trial to be feasible and safe. Within a single session people with SCI can transfer into, achieve autonomous control of, ambulate and exercise within the REX and as a result it may be widely acceptable to this client group. Future research will explore differences between people with tetraplegia and paraplegia and those with complete and incomplete spinal cord lesions, along with patient reported quality of life outcomes following device use. This report lends support to the growing body of evidence that robotic exoskeletons are potentially useful devices for the rehabilitation of people with neurological impairment.

## Abbreviations

ASIA: American Spinal Injuries Association
CA: California
FDA: Federal Devices Agency
GCP: Good Clinical Practice
LCD: Liquid Crystal Display
NHS: National Health Service
NRES: National Research and Ethics Service
OH: Ohio
PLC: Public Limited Company
RAP: Robot Assisted Physiotherapy
RAPPER: Robot Assisted Physiotherapy Exercises in the Rex
REC: Regional Ethics Committee
SAE: Serious Adverse Event
SCI: Spinal Cord Injury
TUG: Timed Up and Go (test)
UK: United Kingdom
USA: United States of America
WMA: World Medical Association
